# An Unusual Finding of the Hyoid Bone

**DOI:** 10.7759/cureus.3365

**Published:** 2018-09-26

**Authors:** Raja Gnanadev, Joe Iwanaga, Marios Loukas, R. Shane Tubbs

**Affiliations:** 1 Miscellaneous, Seattle Science Foundation, Seattle, USA; 2 Medical Education and Simulation, Seattle Science Foundation, Seattle, USA; 3 Anatomy, St. George's University, St. George's, GRD; 4 Neurosurgery, Seattle Science Foundation, Seattle, USA

**Keywords:** hyoid, larynx, ossification, omohyoid, variation, myositis ossificans, forensic pathology

## Abstract

The hyoid contributes to many biomechanical processes including swallowing. Additionally, the hyoid bone has been studied for over a century in an effort to catalog and categorize many observed biometric differences. This has led to the hyoid being a major structure involved in forensic pathology. In this paper, we discuss a very unusual finding of an adult female hyoid bone.

## Introduction

The hyoid bone is a unique structure in the human body for many reasons. The larynx is an extremely cartilaginous area, except for the sole regional bony structure—the hyoid bone. Famously, the hyoid bone is the only bone in humans that does not articulate with any other bone, but only has muscular, ligamentous, and cartilaginous attachments. Given this peculiarity, it has been described as “free floating” [1].

Given that the vast number of muscles is attached to the hyoid, it is no surprise that the hyoid bone contributes to actions such as mastication and swallowing. Additionally, variations in morphometric data between the sexes have been well studied and have had a strong contribution to forensic medicine for decades [2-3].

Herein, we report an unusual variation of the hyoid bone and briefly review morphometric studies on hyoid bone anatomy.

## Case presentation

**Figure 1:**
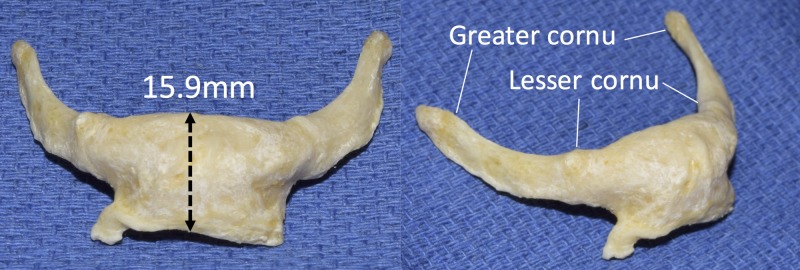
Measurement of the variant hyoid bone.

During the routine dissection of the head and neck of an adult female Caucasian fresh frozen cadaver, an unusual morphology of the hyoid bone was observed. The sternohyoid and omohyoid muscles are attached to the lower border of the hyoid body as usual. The hyoid bone was resected from the anterior neck and the soft tissues on the bone were removed. The height of the body of the hyoid bone then was found to be larger than normal and measured 15.9 mm (Figure 1). However, the greater cornu and lesser cornu were found to be normal in size and orientation. The inferior edge of the body of the bone was drawn out and presented with an unusual edge at the site of attachment of the omohyoid and sternohyoid muscles. No previous scars or evidence of trauma was found in this specimen in the region dissection. Additionally, no other anatomical variations were noted. 

## Discussion

The hyoid bone is located inferior to the mandible, and superior to the thyroid cartilage at the level of the third cervical vertebra in the midline. Laryngeal muscles attach on the superior body, inferior body, and laterally along the each of the horns. Embryologically, the cartilage of the second pharyngeal (hyoid) arch forms the lesser horn and that of the third pharyngeal arch gives rise to the greater horn. The ventral side of the second and third pharyngeal arches fuses to form the body of the hyoid bone during the fourth week of gestation.

This report focuses on the inferior part of the hyoid body where the omohyoid and sternohyoid muscles attach. An elongated body (15.9 mm) along this inferior edge was noted in the specimen.

Many researchers have investigated the sex dimorphism observed regarding the hyoid bone and the variation appears to be consistent across many measurements. These variations include angles of measurement from the body to the greater and lesser tubercles, length of the horns, and thickness of the body, among many others [1-3]. By cataloging these variations, forensic pathologists have been able to use hyoid bone morphology with relatively good accuracy to make determinations related to identifying the deceased [3]. Given the significance of being able to do this, a relatively complex categorization system has been developed for various known shapes of the hyoid. Remarkably, it has been noted that 60% of hyoid bones still do not fit into one of these commonly described categories [4].

Unfortunately, there are few studies that have analyzed the distance from the superior border to the inferior border of the body. A search of the literature has yielded just two studies, separated by over a century, with metrics on this dimension of the hyoid. In 1909, Parsons analyzed the hyoid bone from 108 adult cadavers from male adults (53), female adults (28), and children (27). His results showed an average height of 1.2 cm for males (range: 1.0-1.6 cm), and 1.0 cm for females (range: 0.9-1.2 cm) [5]. In 2012, a group of Japanese researchers analyzed 600 hyoid bones (310 males, 290 females) using three-dimensional computed tomography (CT) imaging. Their data closely matched that of the 1909 study showing a mean height of 9.4 mm in males (range: 6.3-16.0 mm), and 7.8 mm in females (range: 3.0-8.8 mm) [6]. However, the exact morphology of the hyoid bone in these cases was not reported.

Finally, there are reported cases of ossification of the muscles that attach to the hyoid bone, e.g., traumatic and genetic myositis ossificans [7]. A review of the literature yielded a single case report on traumatic myositis ossificans in the superior belly of the omohyoid. In this case, the ossification was unilateral, palpable on physical examination, and clearly visible on radiographs [8].

Anatomical variations such as the one described herein, can result in wrong level surgery as the normally positioned and sized hyoid bone is used by surgeons to estimate the C3 vertebral level [9-12]. Furthermore, as the anterior neck is often palpated during physical examination, bony variants such as the one described here could lead to misdiagnosis or at least, unnecessary testing.

## Conclusions

The hyoid bone has both many unique anatomical features and clinical relevance in forensic pathology. Therefore, reports of hyoid bone variations are important to anatomists and clinicians alike.
